# The Blockade of Interleukin-2 During the Acute Phase of *Trypanosoma cruzi* Infection Reveals Its Dominant Regulatory Role

**DOI:** 10.3389/fcimb.2021.758273

**Published:** 2021-11-17

**Authors:** Jorge Nihei, Fabiola Cardillo, Jose Mengel

**Affiliations:** ^1^ Gonçalo Moniz Research Institute, Oswaldo Cruz Foundation (Fiocruz), Salvador, Brazil; ^2^ Center of Health Sciences, Federal University of Recôncavo da Bahia (UFRP), Santo Antonio de Jesus, Brazil; ^3^ Oswaldo Cruz Institute, Oswaldo Cruz Foundation (Fiocruz), Rio de Janeiro, Brazil; ^4^ Petropolis Medical School, University Faculties Arthur Sa Earp Neto (FMP/UNIFASE), Petropolis, Brazil

**Keywords:** Chagas’ disease, *Trypanosoma cruzi*, regulatory T cells, anti-IL-2, interleukin-2, interleukin-10, interleukin-17, myocarditis

## Abstract

*Trypanosoma cruzi* infection causes Chagas’ disease in humans. The infection activates the innate and adaptative immunity in an orchestrated immune response to control parasite growth, guaranteeing host survival. Despite an effective immune response to the parasite in the acute phase, the infection progresses to a chronic stage. The parasite infects different tissues such as peripheral neurons, the brain, skeletal muscle, and heart muscle, among many others. It is evident now that tissue-specific immune responses may develop along with anti-parasite immunity. Therefore, mechanisms to regulate immunity and to ensure tissue-specific tolerance are operating during the infection. Studying those immunoregulatory mechanisms is fundamental to improve host protection or control inflammatory reactions that may lead to pathology. The role of IL-2 during *T. cruzi* infection is not established. IL-2 production by T cells is strongly down-modulated early in the disease by unknown mechanisms and remains low during the chronic phase of the disease. IL-2 activates NK cells, CD4, and CD8 T cells and may be necessary to immunity development. Also, the expansion and maintenance of regulatory T cells require IL-2. Thus, IL-2 may be a key cytokine involved in promoting or down-regulating immune responses, probably in a dose-dependent manner. This study blocked IL-2 during the acute *T. cruzi* infection by using a neutralizing monoclonal antibody. The results show that parasitemia and mortality rate was lower in animals treated with anti-IL-2. The percentages and total numbers of CD4^+^CD25^+^Foxp3^+^ T cells diminished within three weeks of infection. The numbers of splenic activated/memory CD4 and CD8 splenic T cells increased during the acute infection. T cells producing IFN-γ, TNF-α and IL-10 also augmented in anti-IL-2-treated infected mice. The IL-2 blockade also increased the numbers of inflammatory cells in the heart and skeletal muscles and the amount of IL-17 produced by heart T cells. These results suggest that IL-2 might be involved in the immune regulatory response during the acute *T. cruzi* infection, dampening T cell activation through the expansion/maintenance of regulatory T cells and regulating IL-17 production. Therefore, the IL-2 pathway is an attractive target for therapeutic purposes in acute and chronic phases of Chagas’ disease.

## Introduction

*Trypanosoma cruzi* infection causes Chagas’ disease in humans (Koberle, 1968; Umezawa et al., 2001; Chagas, 2008; Rassi et al., 2010). Parasites in the bloodstream are a characteristic of acute illness. The infection activates the innate and adaptative immunity in an orchestrated immune response to control parasite growth (Brener and Gazzinelli, 1997; Cardillo et al., 2015). Despite an effective immune response to the parasite in the acute phase, the infection progresses to a chronic stage (Cardillo et al., 2015). The parasite infects different tissues such as peripheral neurons, brain, skeletal muscle, and heart muscle, among many others (Rassi et al., 2010). In humans, trypanocidal drugs may clear the parasite if used in the acute infection but seem ineffective in stopping disease progression when given during the chronic illness (Rassi et al., 2010; Morillo et al., 2015). It is evident now that tissue-specific immune responses may develop along with anti-parasite immunity (Leon and Engman, 2003; Lu et al., 2010). Therefore, mechanisms to regulate immunity and ensure tissue-specific tolerance are likely to operate during the infection. These observations suggest that a balanced immune response must be achieved to control the condition and the host survival with minimal tissue damage. Only about 30% of infected people developed clinical disease, arguing that most infected subjects develop an appropriate immune response (Leon and Engman, 2003; Cardillo et al., 2015). So, studying immunoregulatory mechanisms is fundamental to either improve host protection or control inflammatory reactions that may lead to pathology (Hunter et al., 1997; Hori and Sakaguchi, 2004). Cytokines and their pathways are crucial targets for immunotherapies in many immune-mediated pathologies (Hunter et al., 1997; Boyman and Sprent, 2012; Arenas-Ramirez et al., 2015). Many cytokines are necessary for immunity during the acute *T. cruzi* infection (Abrahamsohn and Coffman, 1996; Cardillo et al., 1996; Basso et al., 2004; Bastos et al., 2007; Miyazaki et al., 2010; Roffe et al., 2012). Interleukin (IL)-17, IFN-γ, TNF-α, and IL-10 may be critical to the host survival and infection control (Abrahamsohn and Coffman, 1996; Cardillo et al., 1996; Basso et al., 2004; Bastos et al., 2007; Miyazaki et al., 2010; Roffe et al., 2012). IL-2 may activate NK cells, γδ T cells, CD4, and CD8 T cells and may also be necessary during acute and chronic *T. cruzi* infection (Liao et al., 2013; Mengel et al., 2016).

Furthermore, IL-2 is required to expand and maintain regulatory T cells (Horak, 1995; Furtado et al., 2002; Malek, 2003; Thornton et al., 2004; Liao et al., 2013). The role of Tregs during the acute phase of *T. cruzi* infection is not settled. Some authors have reported that these cells have a modest but significant biological function, as their inactivation improves the host resistance to *T. cruzi* (Kotner and Tarleton, 2007; Mariano et al., 2008; Sales et al., 2008; Nihei et al., 2014; Bonney et al., 2015). However, the studies above have used different monoclonal antibodies to CD25 molecules, interfering directly with the IL-2 axis (Sakaguchi et al., 1995). In this study, we blocked IL-2 using a neutralizing monoclonal antibody (Setoguchi et al., 2005). We further analyzed the frequency of regulatory T cells and the effector conventional T cell pool for their activation status and the secretion of cytokines implicated in the resistance and survival to *T. cruzi* infection as biomarkers for immunoregulation. The results show that parasitemia and mortality rate was lower in mice treated with anti-IL-2. The percentages and total numbers of CD4^+^CD25^+^Foxp3^+^ T cells diminished within three weeks of infection in animals treated with anti-IL-2 mAb. Neutralization of IL-2 increased the numbers of recently activated CD4^+^CD69^+^ T cells and memory CD4 and CD8 T cells after three weeks of infection and the numbers of splenic T cells producing IFN-γ, TNF-α and IL-10. The IL-2 blockade also increased the number of inflammatory cells and T cells secreting IFN-γ, IL-17, and IL-10 in the heart and skeletal muscles. These results suggest that IL-2 might be involved in the immune regulatory response during the acute *T. cruzi* infection, dampening T cell activation and the production of inflammatory cytokines. These functional activities might be related to the expansion/maintenance of regulatory T cell function/numbers, a direct effect of IL-2 on cells that express high-affinity receptors (CD25), as previously shown (Horak, 1995; Furtado et al., 2002; Malek, 2003; Thornton et al., 2004; Liao et al., 2013).

## Materials and Methods

### Animals

C57Bl/6 mice (1-2 months old) were from the Centro de Pesquisas Gonçalo Moniz animal house. The animals were kept in micro-isolators under conventional conditions and were manipulated according to institutional ethical guidelines. The Committee approved all the protocols used in this study for Ethics of the Oswaldo Cruz Foundation, under the protocol number: CpQGM 015-09.

### Infection and Treatment With Anti-IL-2 Monoclonal Antibody

Groups of 5 to 20 mice were infected intraperitoneally with 10^3^ blood-form trypomastigotes of the Tulahuen strain of *T. cruzi* in 0.2 ml of 0.15 M phosphate-buffered saline (PBS). Control mice received the same volume of PBS. The numbers of parasites were evaluated in 5 μL volumes of blood. Anti-IL-2 mAb (clone JES6-1A12) and rat Ig were semi-purified from ascitic fluid or sera, as previously described (Setoguchi et al., 2005; Nihei et al., 2014). Rat Ig has been used as a control in infected mice. A half milligram of the antibody preparations was injected intravenously on days -1, +1, +3, +5, and +7 of the infection.

### Preparation of Heart and Skeletal Muscles Mononuclear Cells

Skeletal muscle or heart fragments were obtained from C57Bl/6 infected mice. Muscles were sliced into small pieces (less than 2 mm diameter) and incubated in collagenase (Sigma-Aldrich) at a 1 mg/ml concentration, diluted in RPMI for 45 min at 37^0^C. Cell suspension and the remaining tissue were further passed through a metal mesh (70 μm pore). Recovered cell suspension was washed three times in incomplete RPMI, and the pellet diluted in 40% Percoll (GE Healthcare Biosciences AB, Uppsala, Sweden). A discontinuous Percoll gradient 40/80% was used to separate mononuclear cells as described before (Dong et al., 2004). Cells at the 40/80 interface were recovered, washed three times in incomplete RPMI, and used further. In addition, cells from different mice in the same group were pooled to allow enough cells for *in vitro* culture and further analysis.

### *In Vitro* Cell Culture

Cells were cultured in triplicates at a density of 10^7^ cells/well in 24-well plates (Nunc) in RPMI 1640 (Gibco, Grand Island, NY) or IMDM (Sigma-Aldrich, Merck, Darmstadt, Germany) supplemented with 10% fetal bovine serum (FBS, Hyclone), 50 mM 2-ME, and 1 mM HEPES (complete medium). In the experiments designed to quantitate IL-17, the cells were cultured in IMDM. Cells were cultured at 37°C and 5% CO_2_ for 24 hours in complete medium alone or in the presence of 2 μg/mL of anti-CD3 monoclonal antibody (clone 2C11). In addition, heart or muscle mononuclear cells were cultured as for the splenic cells (Dong et al., 2004). In some experiments, a combination of PMA plus calcium ionophore was used for cell stimulation. In addition, Brefeldin-A was added 6 hours before the cells were harvested to stain them for flow cytometric analysis.

### Flow Cytometric Analysis

Spleen cells were isolated as described (Cardillo et al., 1993) and placed in ice-cold PBS supplemented with 5% FBS and 0.01% sodium azide. The fluorochrome-conjugated monoclonal antibodies used were anti-CD4 (clone GK1.5), anti-CD8 (clone 53-5.8), anti-CD44 (clone IM7), anti-CD62L (clone MEL-14), anti-CD69 (clone H1.2F3), anti-CD25 (clone PC61), anti-foxp-3 (clone FJK-16s), anti-IL-10 (clone JES5-16E3), anti-IL-17 (clone TC11-18H10.1), anti-IFN-γ (clone XMG1.2) and anti-TNF-α (MP6-XT22) were purchased from eBioscience, Biolegend, or CALTAG. Streptavidin-PE-Cy5.5 revealed biotin-conjugated antibodies from CALTAG. Intracellular staining for IL-10, IL-17, IFN-γ, and TNF-α were performed as described (Cardillo et al., 2007). After surface stainings, live cells were fixed with 1% paraformaldehyde in PBS. All samples were analyzed using a tree colors FACScan (Becton and Dickinson). At least 10^6^ total events were collected per sample. For spleen cells, mice were analyzed individually. For tissue-derived mononuclear cells, a pool of cells from different mice was used in each experiment. Files were analyzed using Flowjo software. An electronic gate was made on lymphocytes using FSCxSSC parameters. Other gates to study activation/memory markers or cytokines were done in CD4^+^ or CD8^+^ T cells.

### Histological and Quantitative Morphological Studies

Heart and skeletal muscle tissues were removed from infected mice and fixed in buffered 10% formalin, paraffin-embedded, and sections used for histological studies. Mononuclear cells or intact parasite nests were counted in 30 non-successive microscopic fields, using a 10x ocular and a 40x objective. In addition, counting was performed on paraffin sections of heart muscle tissues from infected mice during acute infection. The slides were coded, and the studies have done double-blind.

### Statistical Analysis

The results are presented as means ± SD. The significance of differences between the experimental and control groups was determined as described in each figure legend. *P* values below 0.05 were considered significant.

## Results

### The Production of IL-2 by Splenic T Cells Increased at Day Four Post-Infection, Decreasing After That

**Figure 1:**
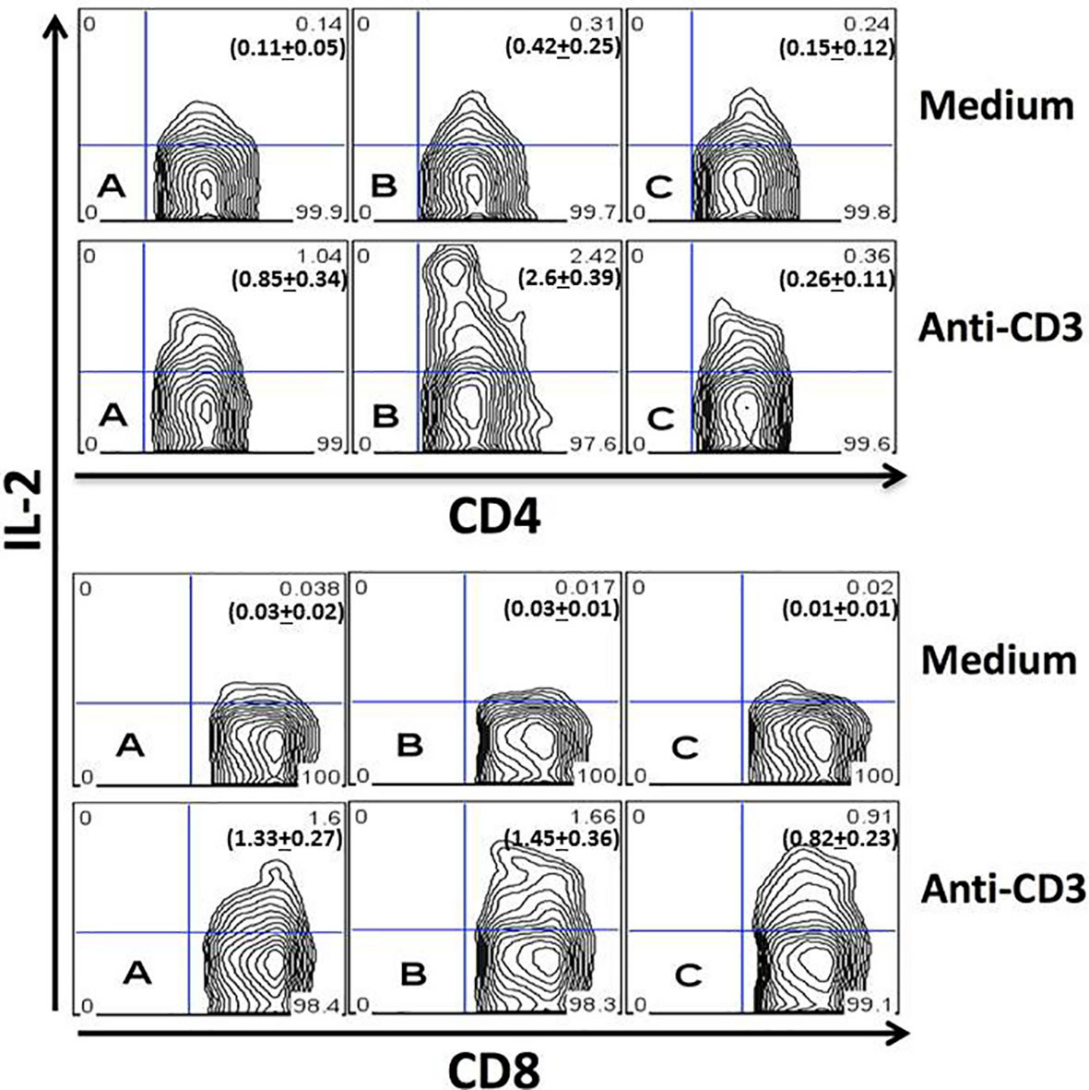
Quantitation of IL-2 production by splenic T cells during acute infection. Spleen cells from uninfected **(A)** or infected mice obtained on the day fourth **(B)** and 21th **(C)** of infection were studied after 24 hours of culture in medium alone (upper plots) or after stimulation with anti-CD3 mAb (lower plots). CD4^+^ (upper panel) and CD8^+^ (lower panel) gated T cells are represented. Each plot represents one mouse from the same experiment. Experiments were repeated three times with similar results. *P* values for IL-2^+^CD4^+^ T cells cultured in medium alone: **(A)** *vs*. **(B)**, P < 0.05; **(A)** *vs*. **(C)**, P > 0.05; **(B)** *vs*. **(C)**, P < 0.05. P values for IL-2^+^CD4^+^ T cells cultured with anti-CD3: **(A)** *vs*. **(B)**, P < 0.001; **(A)** *vs*. **(C)**, P > 0.05; **(B)** *vs*. **(C)**, P < 0.001. P values for IL-2^+^CD8^+^ T cells cultured with anti-CD3: **(A)** *vs*. **(B)**, P > 0.05; **(A)** *vs*. **(C)**, P < 0.05; **(B)** *vs*. **(C)**, P < 0.05. Numbers in bold depicted in the flow cytometry plots represent the mean + SD of each group. ANOVA with Tukey-Kramer’s post-test was used to compare groups of mice, n= 4.

To study the production of IL-2 by T cells during the acute *T. cruzi* infection, spleen cells from uninfected mice (**Figure 1**, plot A) or infected mice obtained on days 4 (**Figure 1**, plot B) and 21 (**Figure 1**, plot C) after infection were cultured for 24 hours in the presence of medium alone (upper charts) or anti-CD3 mAb (lower charts). CD4^+^ (**Figure 1**, representative upper plot panel) or CD8^+^ (**Figure 1**, representative lower plot panel) gated T cells are shown in **Figure 1**. The results demonstrate an augmented percentage of IL-2^+^ CD4^+^ T cells on day four after infection compared to days 0 or 21 with or without stimulation with anti-CD3 mAb. In addition, the percentage of IL-2^+^ CD4^+^ T cells upon anti-CD3 stimulation was decreased compared to day 0 or day four post-infection. We did not find increased percentages of IL-2 producing CD8^+^ T cells at any moment after infection. On the contrary, a decreased percentage of IL-2^+^ CD8^+^ splenic T cells on day 21 after infection compared to days 0 or 4 was demonstrated.

### *In Vivo* Treatment With Blocking Antibody to IL-2 in C57Bl/6 Mice Infected With the Tulahuen Strain of *T. cruzi* Resulted in Lower Parasitemia and Higher Survival Rates

**Figure 2:**
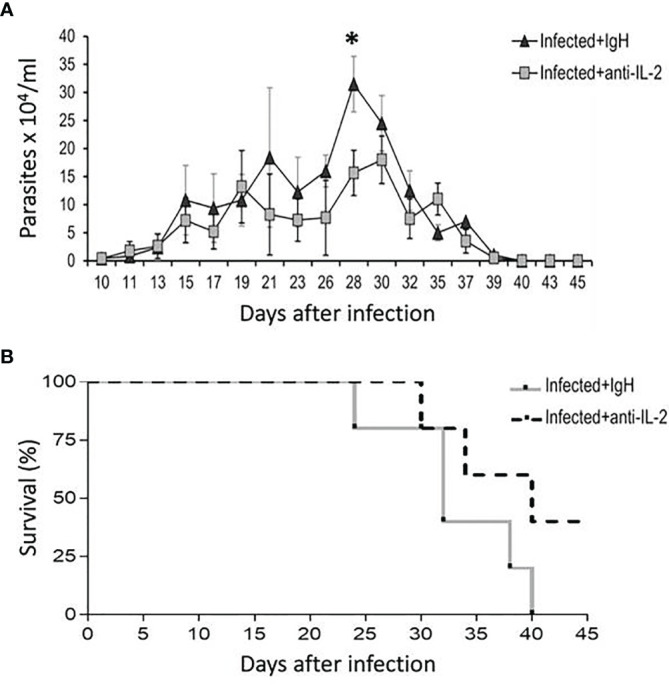
Parasitemia and survival rates of mice treated with blocking antibody to IL-2 or rat Ig in C57Bl/6 infected mice. The number of parasites in the bloodstream of C57Bl/6 mice treated with blocking mAb to IL-2 or rat Ig is shown in panel **(A)**. Statistical significance was observed only at a one-time point, at the parasitemia peak (28 days after initial infection). Mann-Whitney test was used to compare different groups of mice (*P < 0.05). Panel **(B)** shows that survival rates were higher in the group treated with anti-IL-2 mAb compared to infected controls treated with rat Ig (P < 0.05, n = 10 at the beginning of the experiment, Gehan-Breslow-Wilcoxon test).

C57Bl/6 mice treated with blocking mAb to IL-2, starting one day before infection, presented lower parasitemia on the 28^th^ day than mice treated with rat Ig as a control (**Figure 2A**). C57Bl/6 mice infected with the Tulahuen strain of *T. cruzi* are extremely susceptible, and 100% of the mice died in the group treated with rat Ig up to day 40 of infection. On the other hand, 40% of the mice were alive by day 40 after infection in the group treated with anti-IL-2 mAb, and these mice survived more than six months (**Figure 2B** and data not shown).

### *In Vivo* Treatment With Anti-IL-2 mAb Led to An Increased Number of Early Activated CD4^+^CD69^+^ T Cells During the Acute Phase of *T. cruzi* Infection in C57BL/6 Mice

**Figure 3:**
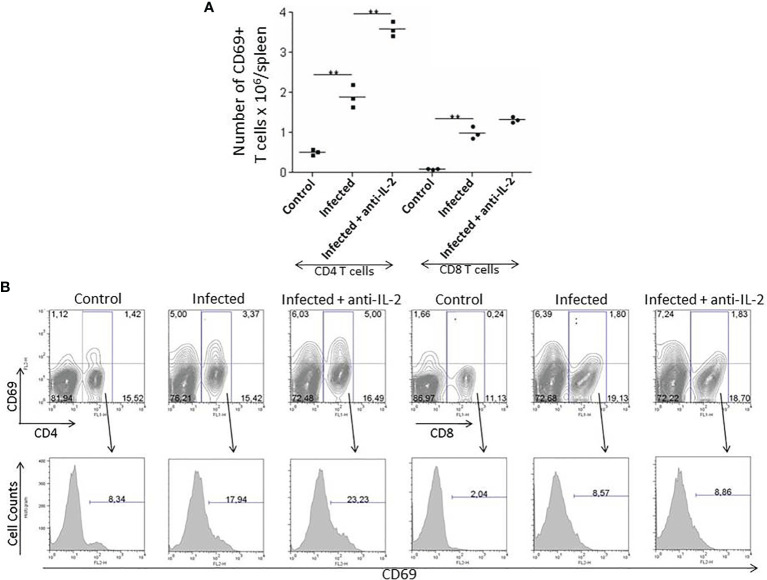
IL-2 blockade leads to increased numbers of early activated splenic CD69^+^ T cells during the acute phase of *T. cruzi* infection in C57BL/6 mice. Panel **(A)** shows the total numbers of splenic early activated CD4+ and CD8+ T cells bearing the CD69 molecule 21 days after infection. Anti-IL-2 treated animals were compared to infected or uninfected control mice treated with rat Ig. Panel **(B)** shows one representative experiment with FACS plots from 3 pooled spleen cells per group of mice. Histograms represent the expression of CD69 in gated CD4^+^ or CD8^+^ splenic T cells. Experiments were repeated on three occasions with similar results (**P < 0.01, n = 3 group, Mann-Whitney test).

The numbers of activated T cells increased during the acute phase *of T. cruzi* infection. The expression of CD69 molecules reveals early T cell activation. **Figure 3A** shows that the total numbers of splenic CD4^+^CD69^+^ T cells increased by day 21 after the initial infection. The increment was higher in mice treated with anti-IL-2 mAb than infected control mice treated with rat IgG.

CD8**^+^** CD69**^+^** splenic T cells were increased in infected mice irrespectively of treatments. However, their total numbers did not differ between infected control (rat IgG treated) or anti-IL-2 mAb treated mice. **Figure 3B** shows one representative experiment with flow cytometry plots from 3 pooled spleen cells per group of mice. Histograms represent the expression of CD69 in gated CD4**^+^** or CD8**^+^** splenic T cells.

### Increased Numbers of Central (CD44^high^CD62L^high^) and Effector (CD44^high^CD62L^low^) Memory T Cells Accompany Anti-IL-2 mAb Treatment During the Acute Phase of *T. cruzi* Infection

**Figure 4:**
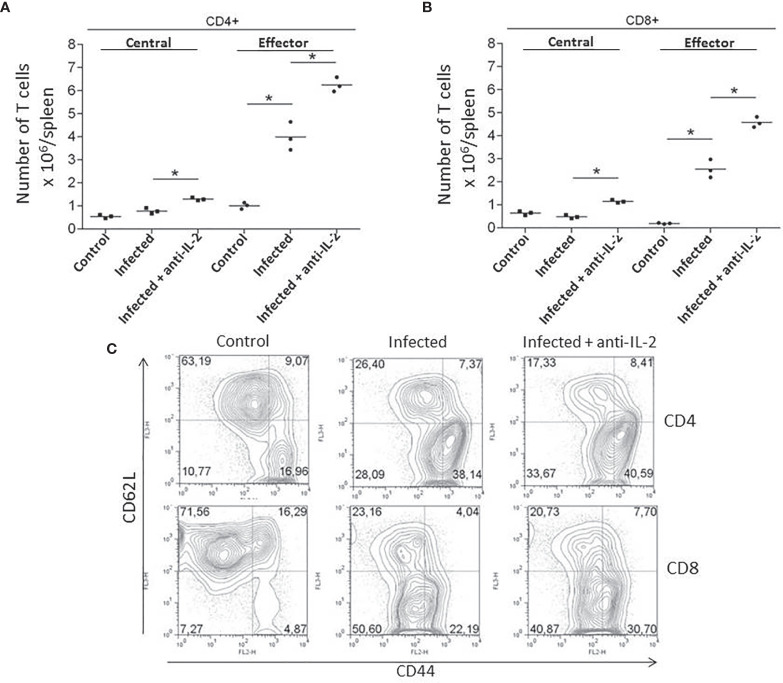
IL-2 blockade increased the numbers of central (CD44^high^CD62L^high^) and effector (CD44^high^CD62L^low^) memory T cells during the acute phase of *T. cruzi* infection. Total numbers of splenic CD4^+^ **(A)** and CD8^+^ **(B)** central (CD44^high^CD62L^high^) and effector (CD44^high^CD62L^low^) memory T cells are compared to infected or uninfected control rat Ig-treated mice. Illustrative FACS plots from one representative experiment, where three pooled spleen cells per group of mice were analyzed, are shown in panel **(C)** (gated CD4^+^ T cells, upper plots and CD8^+^ T cells, lower plots). These experiments were repeated three times with similar results (*P < 0.05, n = 3 group, Mann-Whitney test).

As the numbers of activated/memory T cells were previously related to resistance in another experimental model of *T. cruzi* infection (Cardillo et al., 2002), we asked if the administration of blocking monoclonal antibodies to IL-2 could somehow induce higher numbers of memory T cells. In fact, *in vivo* treatment with anti-IL-2 resulted in increased numbers of both splenic CD4^+^ (**Figure 4A**) and CD8^+^ (**Figure 4B**) central (CD44^high^CD62L^high^) and effector (CD44^high^CD62L^low^) memory T cells when these mice were compared to control rat IgG treated mice. Illustrative flow cytometry plots from one representative experiment are shown in **Figure 4C** (gated CD4+ T cells) and 4D (gated CD8+ T cells).

### The Numbers of Splenic T Cells Producing IFN-γ, IL-10, and TNF-α Were Increased in Anti-IL-2 Treated Mice During the Acute Phase of the Infection

**Figure 5:**
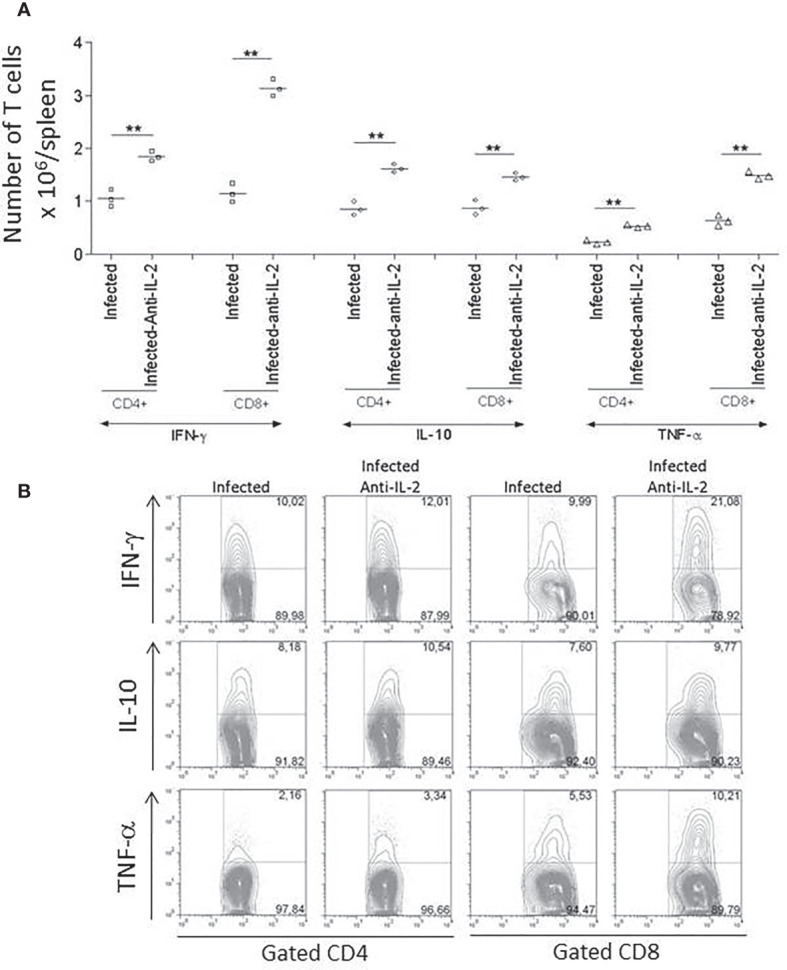
Total numbers of splenic T cells producing IFN-γ, IL-10, and TNF-α were increased in infected mice treated with anti-IL-2 mAb. Panel **(A)** shows the numbers of splenic CD4^+^ and CD8^+^ T cells producing IFN-γ, IL-10, and TNF-α compared to infected control groups that received rat Ig. The results of three individual mice per group are shown. The FACS plot panel **(B)** represents one experiment demonstrating gated CD4^+^ and CD8^+^ splenic T cells from 3 to 4 pooled spleens from infected rat Ig- or anti-IL-2-treated mice stained for intracellular cytokines as indicated. Spleen cells were stimulated with anti-CD3 for 24 hours and brefeldin-A for the last six hours. The experiment represents a total of three studies with similar results (**P < 0.01, n = 3 group, Mann-Whitney test).

The production of IFN-γ and TNF-α during the acute phase of the infection was previously related to resistance to *T. cruzi* infection (Abrahamsohn and Coffman, 1996). For this reason, we asked if the treatment with anti-IL-2 was modulating the overall production of these cytokines. **Figure 5** shows that the *in vivo* treatment with JES6-1A12 monoclonal antibody increased the total splenic CD4^+^ and CD8^+^ (**Figure 5A**) T cells that produced IFN-γ and TNF-α when compared to control groups that received rat IgG. In addition, we have also observed an increase in the total numbers of CD4^+^ and CD8^+^ T cells that produced IL-10 (**Figure 5A**). The flow cytometry plot panel (**Figure 5B**) is from one representative experiment demonstrating gated CD4^+^ and CD8^+^ splenic T cells from control infected rat IgG or anti-IL-2 treated mice, stained for intracellular cytokines.

### *In Vivo* Administration of Anti-IL-2 mAb Diminishes CD4^+^CD25^+^ T Cells and Decreases the Percentage and Numbers of Splenic CD4^+^CD25^+^Foxp3^+^ T Cells

**Figure 6:**
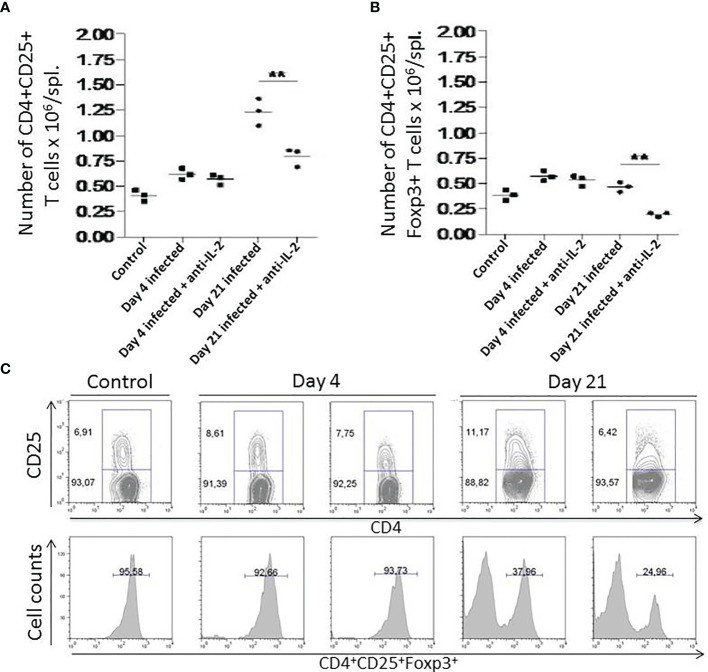
Numbers of splenic CD4^+^CD25^+^ and CD4^+^CD25^+^Foxp3^+^ T cells after *In vivo* administration of anti-IL-2 mAb. In **Figure 6**, total numbers of splenic CD4**^+^** CD25**^+^ (A)** and CD4**^+^** CD25**^+^** Foxp3**^+^** T cells **(B)** are shown. Days 4 and 21 after infection are compared with uninfected controls. Mice that received anti-IL-2 mAb treatment were compared with uninfected or infected mice treated with rat Ig. Rat Ig treatment did not produce differences concerning control PBS-treated mice (not shown). **(C)** (upper panel) shows representative plots of gated splenic CD4**^+^** CD25**^+^** T cell population after *in vivo* treatment with anti-IL-2 mAb treated mice after different time-points post-infection compared to controls. Representative histograms (**C**, lower panel) from 3 pooled spleen cells per group of mice show the percentage of Foxp3^+^ cells inside gated CD4^+^CD25^+^ T cells compared in distinct time-points after infection. Experiments were repeated on three occasions with similar results (**P < 0.01, n = 3 group, Mann-Whitney test).

In **Figure 6**, we have compared the *in vivo* activity of anti-IL-2 mAb with rat IgG administration during *T. cruzi* acute infection; **Figures 6A, B** show a strong reduction in total numbers of splenic CD4**^+^** CD25**^+^** and CD4**^+^** CD25**^+^** Foxp3**^+^** T cells on day 21 after infection upon IL-2 blockade. Control and Infected mice were treated with rat IgG. Previous experiments showed that this scheme of rat IgG treatment did not affect the numbers of splenic CD4**^+^** CD25**^+^** Foxp3**^+^** T cells in infected or non-infected mice (data not shown). **Figure 6C** (upper panel) shows that anti-IL-2 mAb could drop the CD4**^+^** CD25**^+^** T cell population; this was especially impressive for the CD25**^+^** high subpopulation 21 days after infection. The percentage of Foxp3+ cells inside gated CD4**^+^** CD25**^+^** T cells has diminished by half of the numbers found in infected mice treated with rat IgG as a control within 21 days after infection (**Figure 6C**, lower panel).

### *In Vivo* Administration of Anti-IL-2 mAb Augments Heart Tissue Inflammation and the Numbers of CD4^+^ T Cells Producing IL-17

**Figure 7:**
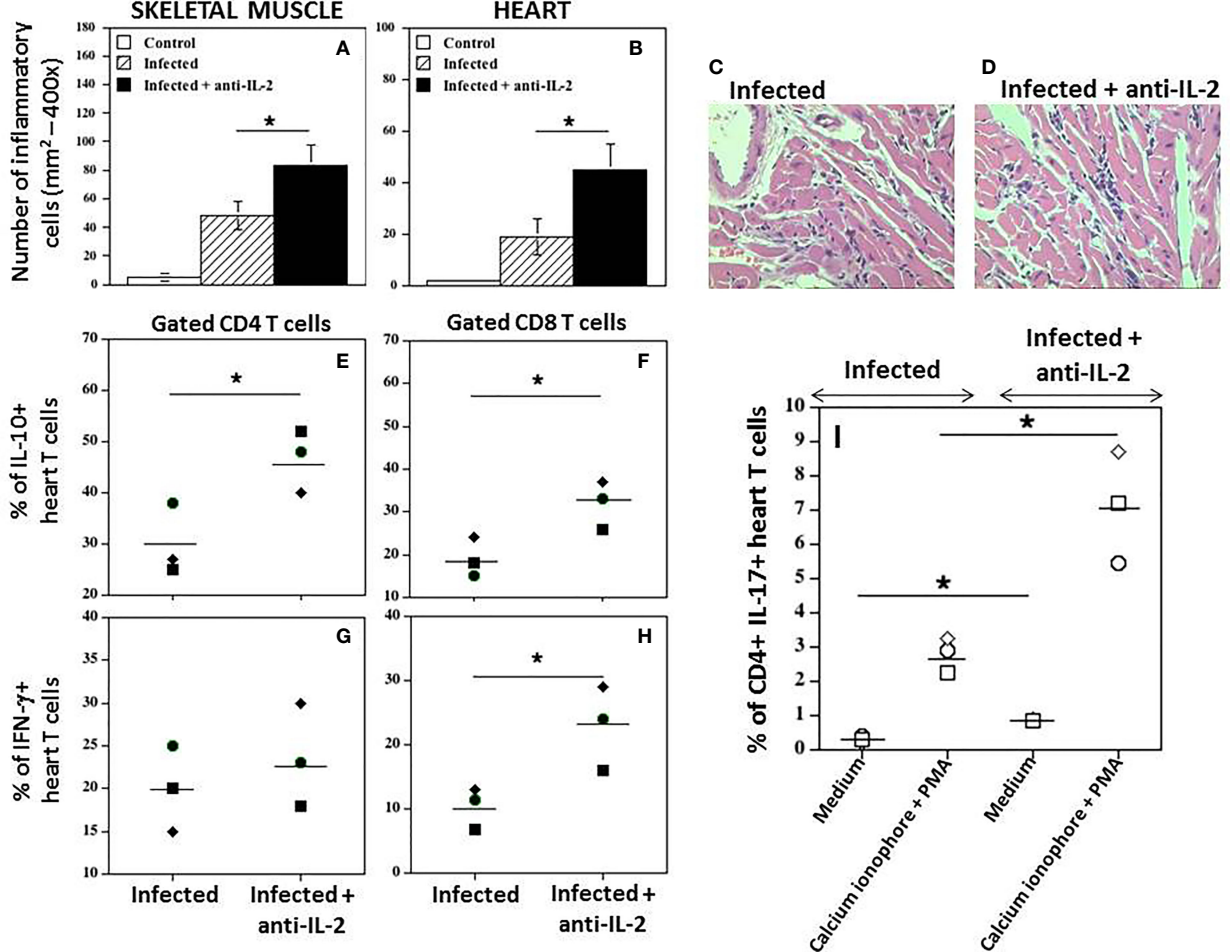
*In vivo* administration of anti-IL-2 mAb augments heart tissue inflammation and the numbers of heart T cells producing IFN-γ, IL-10, and IL-17. **Figure 7** shows the number of inflammatory cells in mice treated with anti-IL-2 in the skeletal muscle **(A)** and heart **(B)**, compared to uninfected or infected control mice treated with rat Ig (*P < 0.05, n = 5-7 group, Mann-Whitney test). Representative heart sections from Ig-treated and anti-IL-2 treated C57Bl/6 infected mice are shown in panels **(C, D)**, respectively, using a 40x objective. Panels **(E–H)** show the percentage of heart CD4+ and CD8+ T cells producing IL-10 and IFN-γ, respectively, compared to the infected control group treated with rat Ig. Tissue-derived cells were stimulated with anti-CD3 mAb for 24 hours, followed by brefeldin-A for the last 6 hours. Panel **(I)** shows the percentage of heart CD4+ T cells producing IL-17 in infected mice treated with anti-IL-2 mAb compared to infected controls injected with rat Ig. In this experimental set, cells were incubated with complete medium alone or activated with Cell Stimulation Cocktail for 36 hours, followed by brefeldin-A for the last 6 hours. (PMA plus ionomycin, Tonbo Biosciences, San Diego, CA, USA). All tissues were removed after 21 days of infection. Each symbol represents one independent experiment done with pooled cells from 3 to 5 mice per group. (*P < 0.05, Mann-Whitney test).

A quantitative study of the inflammatory infiltrate presented in the heart, and striated muscle was performed. **Figure 7** shows that the number of inflammatory cells in the skeletal muscle (A) or heart (B) was significantly higher in infected mice treated with anti-IL-2 mAb when compared to control mice treated only with rat IgG. In addition, although not statistically significant, the numbers of parasite nests tended to be lower in the group treated with anti-IL-2 (data not shown), suggesting that a potentiated immune response developed despite parasite tissue load. **Figures 7C, D** illustrate, in high magnification, heart sections from Ig-treated and anti-IL-2 C57Bl/6 infected mice, respectively. Please note the larger inflammatory foci in the heart of animals treated with anti-IL-2.

Interestingly, IL-10**^+^** CD4**^+^** (**Figure 7E**) and IL-10**^+^** CD8**^+^** (**Figure 7F**) heart T cells were increased in mice treated with anti-IL-2 mAb during this phase of the infection compared to control infected rat IgG-treated mice. In **Figure 7G**, it is demonstrated that the percentage of CD4 T cells from heart tissues producing IFN-γ was not different compared to the infected control group. However, the amount of CD8**^+^** heart T cells producing IFN-γ was higher in the group treated with anti-IL-2 (**Figure 7H**). In addition, the percentage of heart CD4^+^ T cells expressing IL-17 was augmented under IL-2 blockade, as depicted in **Figure 7I**. The increased frequency of CD4^+^IL-17^+^ cells in the heart of mice treated with anti-IL-2 was further potentiated by using a short period of stimulation with PMA and calcium ionophore (**Figure 7I**).

## Discussion

*Trypanosoma cruzi* infects many cell types and mainly produces pathology in the digestive tube’s neuronal tissues, heart muscle, and intracardiac neurons (Rassi et al., 2010). The pathology is associated with tissue inflammation that, on the one hand, helps curb infection but, on the other hand, induces direct or bystander tissue damage mainly in the acute phase of the disease where parasites are abundant (Cardillo et al., 2015). Once tissue infection is controlled to very low levels, the immune response checks parasite numbers (Ward et al., 2020). At this early chronic stage, tissue inflammation is not evident but limited to focal inflammatory infiltrates with or without low numbers of parasites (Mengel and Rossi, 1992). Secondary tissue damage at the early chronic disease is less important than acute infection, but it may provoke chronic tissue lesions (Chatelain, 2017; Chadalawada et al., 2020). The host can survive for years (humans) or months (mice), or even for the entire life without showing any signs of organ failure or only minimal clinical and laboratory findings (Rassi et al., 2010). This condition is called indeterminate chronic disease (Chadalawada et al., 2020). However, in about 30% of infected human subjects or some mice/parasite strain combinations, a conspicuous heart and skeletal muscle inflammation with severe damage and fibrosis develop at the disease’s chronic stage (Rassi et al., 2010). Parasite load remains at very low levels in either symptomatic or asymptomatic hosts, arguing that other factors related to the host may have a role in determining pathology *versus* non-pathology (Cunha-Neto et al., 2011; Bonney and Engman, 2015; Mengel et al., 2016). Although some cases of expontaneous cure of Chagas disease are suggested, definitive proof on this issue lacks (Dias et al., 2008). Therefore, a lifetime chronic infection is a rule. Available trypanocidal drugs are only effective in the early phases of the disease and did not benefit established symptomatic chronic patients (Morillo et al., 2015). In addition to more efficient and less toxic trypanocidal drugs (Rao et al., 2019), treatments aiming at the host factors to limit tissue damage are needed (Nihei et al., 2014; Mengel et al., 2016). Therefore, it is crucial to understand the inflammatory tissue process that may or may not induce tissue damage and fibrosis during the acute and chronic phases of the disease (Lewis et al., 2014; Lewis et al., 2018).

Many cells and cytokines are involved in the acute and chronic immune response and tissue inflammatory process during *T. cruzi* infection (Meyer Zum Buschenfelde et al., 1997; Ferreira et al., 2003; Cardillo et al., 2004; Fiuza et al., 2009; Nogueira et al., 2014; Pack and Tarleton, 2020). In addition, it is known that cells from the innate and adaptative immune response are activated during acute illness and remain activated for long periods after that (Cardillo et al., 2004; Sathler-Avelar et al., 2009). Therefore, cells from the adaptative immunity are fundamental to restrain infection in the tissues by producing inflammatory cytokines such as IFN-γ, TNF-α, and IL-17 (Abrahamsohn and Coffman, 1996; Cardillo et al., 1996; Basso et al., 2004; Bastos et al., 2007; Miyazaki et al., 2010; Roffe et al., 2012). On the other hand, the production of IL-10 and the activity of regulatory T cells may downregulate the inflammatory process, limiting tissue damage and perhaps the effectiveness of parasite control in some infections (Hunter et al., 1997; Sakaguchi, 2004; Roncarolo et al., 2018). A delicate balance between these two arms of the immune response might favor the host survival in the expenses of sterile immunity (Hunter et al., 1997; Sakaguchi, 2004; Roncarolo et al., 2018). Different populations of regulatory T cells have been described so far (Sakaguchi et al., 2006; Ligocki and Niederkorn, 2015). In many experimental systems, the activity of CD4^+^CD25^+^Foxp3^+^ Treg cells has a major impact on immune system regulation. It has been shown that a lower number of Treg cells is found in Chagasic patients that have cardiomyopathy when compared to infected patients without clinical signs of heart disease (de Araujo et al., 2012). In mice, the picture is less clear. Evidence favoring an important role for Tregs in acute infection exists (Bonney et al., 2015; Araujo Furlan et al., 2018), but other studies show minimal activity (Kotner and Tarleton, 2007; Sales et al., 2008). These controversies could be due to different experimental designs. Also, the use of monoclonal antibodies to CD25 may affect other effectors and activated cells that may express this molecule, making the results open to different interpretations.

CD4^+^CD25^+^Foxp3^+^ Treg cells depend on the presence of IL-2 to expand and perform their regulatory functions (Horak, 1995; Furtado et al., 2002; Malek, 2003; Thornton et al., 2004; Liao et al., 2013). *In vivo* administration of blocking monoclonal antibodies to IL-2 diminishes the total number of Treg cells by depleting IL-2 bioactivity, causing autoimmune diseases (Setoguchi et al., 2005). Therefore, IL-2 blocking antibodies allow us to take a different approach to investigate the IL-2/Treg axis, previously proposed as a target for host-centered clinical interventions (Mengel et al., 2016). Previous studies have shown that the production of IL-2 is downregulated during the acute phase of *T. cruzi* infection in mice and humans (Briceno and Mosca, 1996; Tarleton, 1988). Our results confirm that the frequency of CD4^+^ and CD8^+^ T cells producing IL-2 drops along with the acute illness, but a peak of IL-2 production on day four after infection preceded the decay (**Figure 1**). Based on our data, we have decided to start administering anti-IL-2 blocking antibody on day -1 and extend the treatment for the first week after infection, therefore avoiding the *in vivo* IL-2 production peak. This treatment resulted in lower parasitemia and lower mortality of anti-IL-2 treated mice (**Figure 2**). These results suggested that IL-2 was involved in mechanisms that could be related to susceptibility and resistance.

Resistance to *T. cruzi* infection is due, among other factors, to a faster production of activated/effector/memory T cells along with the acute infection (Cardillo et al., 2002). CD69 is an early marker presented in recently-activated T cells (Hamann et al., 1993). FACS analysis of splenic T cells concerning the presence of CD69 showed that mice treated with anti-IL-2 mAb had increased numbers of CD4^+^CD69^+^ splenic T cells 21 days after initial infection (**Figure 3**). We could also see a tendency (non-statistically significant) of having increased numbers of recently activated splenic CD8^+^ T cells in the groups treated with anti-IL-2 mAb. A similar picture for central and effector memory T cells of either CD4^+^ or CD8^+^ subsets could be observed. Inside both T cell subsets, there was an increased number of central and effector splenic memory T cells 21 days after infection in the group treated with anti-IL-2 mAb (**Figure 4**). Therefore, IL-2 blockade induced the formation of a larger set of recently activated and memory T cells compared to control rat Ig-treated infected mice. These larger numbers of activated/memory T cells may reflect functional differences that may translate into the potentiation of cytokine patterns such as Th1 or Th2, influencing the outcome of the infection (Locksley and Scott, 1991; Hoft et al., 2000; Stockinger et al., 2006). As far as one is concerned, there is no clear bias in the T cell cytokine patterns (Th1 or Th2) correlated with susceptibility or resistance during *T. cruzi* infection (Hoft et al., 2000). However, some inflammatory cytokines, namely IFN-γ and TNF-α, are closely related to better-controlling parasite growths (Abrahamsohn and Coffman, 1996; Cardillo et al., 1996). In addition, IL-10 has been associated with better survival and control of parasite numbers in some models of *T. cruzi* infection (Roffe et al., 2012). These surprisingly biological effects probably allow higher tissue levels of inflammatory cytokines, limiting collateral tissue damage or possibly directly impacting CD8 T cell activation improvement, as previously suggested (Hunter et al., 1997; Guo et al., 2021). Our study provides evidence that IL-2 dampened the production of IFN-γ, TNF-α, and IL-10 by splenic T cells, as shown in **Figure 5**. These data, all together, indicate that IL-2 has a dominant down regulatory role during the acute phase of *T. cruzi* infection either in the development of an activated/memory T cell response or in the frequency of cells producing inflammatory and non-inflammatory cytokines. As previously pointed out, IL-2 may be crucial for the expansion and function of Tregs (CD4+CD25+Foxp3+) in peripheral lymphoid tissues (Boyman et al., 2006; Boyman and Sprent, 2012). Lack of IL-2 or their different receptor chains produces T cell hyperactivation and the development of various autoimmune disorders (Horak, 1995; Schimpl et al., 2002). Our data showed that upon anti-IL-2 mAb administration, mice had fewer CD4^+^CD25^+^Foxp3^+^ T cells at day 21 post-infection (**Figure 6B**). Therefore, the decay of Treg numbers may well justify the greater increase of the numbers of activated/memory T cells and the increased frequency of T cells producing different cytokines. The new balance created by the precocious inactivation of IL-2 may influence the outcome of the disease with better control of parasite numbers and improved survival in the acute phase of the infection.

As discussed above, the inflammatory process is an important step of the immune response that clears tissue infection. We have previously observed that blocking CD25 with a nondepleting mAb may curb inflammation in the early chronic phase of *T. cruzi* infection (Nihei et al., 2014). We and others have hypothesized that blocking CD25 would favor the accumulation of bioactive IL-2 and a subsequent increase in Treg cells that, in turn, would downregulate the inflammatory reaction (Nihei et al., 2014; Huss et al., 2015). So, the game played by IL-2 and Tregs would be very sensitive to possible therapeutic manipulations by increasing or decreasing the availability of IL-2 in many different ways (Boyman et al., 2006; Arenas-Ramirez et al., 2015; Chen et al., 2020; Khoryati et al., 2020; Solomon et al., 2020). Therefore, we hypothesized that blocking IL-2 during the initial phases of T. cruzi infection would also cause modifications in the inflammatory reaction. **Figures 7A, B** showed that blockade of IL-2 during acute infection potentiates inflammation by increasing the numbers of mononuclear cells in the heart and skeletal muscle tissues. In addition, the frequencies of T cells secreting IFN-γ and IL-10 were increased within 21 days of infection, as demonstrated in **Figures 7E–H**.

Furthermore, the blockage of IL-2 led to an increased frequency of Th17 cells in the heart, which may contribute not only to control tissue parasitism but also to potentiate tissue lesions (Miyazaki et al., 2010; Erdmann et al., 2013; Kitada et al., 2017). IL-2 downregulates the production of IL-17 either directly through STAT5 (Laurence et al., 2007) or indirectly by inhibiting the IL-23 activity on Th17 cells and, therefore, IL-2 blockade may directly correlate with higher IL-17 production (Stockinger, 2007; Dai et al., 2017; Chen et al., 2021). However, IL-2 may not be directly responsible for the control of IFN-γ and IL-10 production. Recently, IL-27 was described as a new important player during intracellular parasitic infections (Kastelein et al., 2007; Cobb and Smeltz, 2012). For instance, IL-27 limits IL-2 production during Th1 differentiation (Owaki et al., 2006). By diminishing IL-2, interleukin-27 could also inhibit the expansion of Treg cells, allowing the development of a stronger Th1 response and higher production of IFN-γ (Owaki et al., 2006). IL-2 production is profoundly suppressed during conventional mice’s acute *T. cruzi* infection, and a marked Th1 response is usually observed (Tarleton, 1988; Cardillo et al., 2004). Artificial IL-2 blocking earlier in the course of acute infection resulted in the potentiation of IFN-γ production, which parallels a lower frequency and numbers of CD4+ Foxp3+ Treg cells, as shown herein. Altogether, these data suggest that IL-27 may improve Th1 responses indirectly by limiting IL-2 production and Treg expansion. However, the IL-27 receptor is also expressed on Tregs, and IL-27 may stabilize Treg functions helping to control T cell over-reactivity in situations where Tregs are in relatively high numbers (Do et al., 2017).

Interestingly, inhibition of IL-2 activity in *T. cruzi* infected mice was followed by increased levels of IL-10 produced by splenic and heart-derived T cells (**Figures 5** and **7**). There is no previous indication that IL-2 could control the production of IL-10 by T cells, but mice that lack IL-27 receptor (IL-27ra-/-) on Tregs develop a more severe autoimmune condition accompanied by higher levels of T cells’ IL-10 production (Do et al., 2017). In conclusion, IL-27 and IL-2 may work in conjunction to set up the numbers and function of Foxp-3+ Treg cells during acute infections. In more limited Treg numbers (absence of IL-2), the resulting IL-27 activity would favor hyperactivation, whereas IL-27 would stabilize and potentiate Treg activity on a Treg sufficiency scenario. Although the nature of the argument considering the interplay between IL-2 and IL-27 is completely theoretical and is devoided of experimental evidence, it remains an open question, easily testable.

In conclusion, this study and others strongly suggest that the IL-2 pathway is an attractive target for manipulating Treg activity in Chagas’ disease.

## Data Availability Statement

The raw data supporting the conclusions of this article will be made available by the authors, without undue reservation.

## Ethics Statement

The animal study was reviewed and approved by Gonçalo Moniz Research Institute, Fiocruz-BA.

## Author Contributions

JN, FC, and JM contributed to conception, and design of the study. JN and JM organized the database. JN performed the statistical analysis. JM wrote the first draft of the manuscript. JN, FC, and JM wrote sections of the manuscript. All authors contributed to manuscript revision, read, and approved the submitted version.

## Conflict of Interest

The authors declare that the research was conducted in the absence of any commercial or financial relationships that could be construed as a potential conflict of interest.

## Publisher’s Note

All claims expressed in this article are solely those of the authors and do not necessarily represent those of their affiliated organizations, or those of the publisher, the editors and the reviewers. Any product that may be evaluated in this article, or claim that may be made by its manufacturer, is not guaranteed or endorsed by the publisher.
